# Exploring and Supporting Parents’ Stories of Loss in the NICU: A Narrative Study

**DOI:** 10.1177/10497323231201023

**Published:** 2023-11-07

**Authors:** Jenna Lakhani, Cheryl Mack, Diane Kunyk, Michael van Manen

**Affiliations:** 1 Department of Pediatrics, 3158 University of Alberta, Edmonton, AB, Canada; 2 Department of Anesthesiology and Pain Medicine, 3158 University of Alberta, Edmonton, AB, Canada; 3 John Dossetor Health Ethics Centre, 3158 University of Alberta, Edmonton, AB, Canada; 4 Faculty of Nursing, University of Alberta, Edmonton, AB, Canada

**Keywords:** bereavement, grief, ethics, moral perspectives, infants, narrative

## Abstract

Death is no stranger to the neonatal intensive care unit (NICU). Extreme prematurity, congenital abnormalities, and other complexities can turn what was hoped to be a very exciting moment in a family’s life into one of despair and grief. There are many infants that not only do not survive but also have a medicalized death necessitating complex decision-making, weighing quality versus duration of life. We can learn from the stories of parents who chose palliative care for their children. In this narrative inquiry study, we elicited bereaved parents’ stories and reflections on the lives of their children and the care they received in the NICU. From a narrative ethics perspective, their stories speak to normative aspects of parenting, decision-making, and receiving medical care that affect their moral sense-making of their NICU experiences as well as their longer-term living with the loss of their children. Their stories express the importance of having had meaningful time with their children, maintaining direct and frequent communication, acknowledging uncertainty, and emphasizing compassion as methods of providing support to parents as they navigate their bereavement.

## Introduction

With the high acuity seen in neonatal intensive care units (NICUs), death is an inevitable phenomenon. Most of these deaths occur following a decision to withhold or withdraw life-sustaining medical interventions, which differs from the general pediatric population where death more often occurs despite escalating intensive care (Richards et al., 2018; Verhagen et al., 2010). With advancements in healthcare technologies, the survival of extremely premature infants or those with significant medical and/or surgical issues is improving; therefore, quality-of-life considerations are moving to the forefront (Currie et al., 2016). These complexities create significant challenges in decision-making as the burden of hospitalization or permanent medical issues may be perceived as less favorable relative to intensive care’s anticipated benefits (Verhagen et al., 2010).

Using a shared decision-making model is the gold standard for approaching medical intervention, end-of-life, and other consequential discussions (Shaw et al., 2020). There are, however, various difficulties that healthcare practitioners face when engaging in conversations surrounding goals of care such as diagnostic uncertainty, prognostic ambiguity, and differing sociocultural beliefs of parents and practitioners (Dombrecht et al., 2020). The most appropriate manner to deliver information in an unbiased manner with well-defined choices based on possible outcomes remains unclear (Janvier et al., 2014), likely reflecting in part the manifold of different lived experiences that these families have lived (van Manen, 2021).

People seem to think about the value of preterm infants’ lives differently compared to those born at term, independent of predicted survival and developmental outcomes (Janvier et al., 2008). For instance, preterm infants are more likely to receive in-hospital cardiopulmonary resuscitation shortly before death compared to mature infants, despite having a higher-associated mortality (Fry et al., 2020). Psychosocial factors within the healthcare team may contribute to this finding, adding to complexities in end-of-life decision-making in the NICU. Although national guidelines exist for decision-making, physicians are often unaware of how to implement them into practice, and personal opinions may differ (Shaw et al., 2016).

The perspectives of parents who ultimately chose palliative goals of care are relatively absent from the NICU literature such that there is a need for research that forefronts their experiences. Supporting parental bereavement is an essential aspect of holistic NICU care, yet understanding how to assist parents to reach their goals and support their bereavement is incomplete. This is in the face of knowing that parents may face a heavy burden of infant loss despite healthcare providers’ support through shared decision-making approaches, memory-making strategies, or intricate communication techniques (Abraham and Hendriks, 2017; Armentrout, 2009; Currie et al., 2016). More so, in a recent systematic review exploring strategies for healthcare practitioners to support parental bereavement, the ability of parents to spend time caring for their child, their perception of infant suffering, their communication experiences with healthcare providers, and their access to alternative means of support such as palliative and spiritual care appeared to be suboptimal (Lakhani et al., 2023). In the included literature, parents described inconsistently being supported to hold their child, to participate in their daily care as parents, and to share their thoughts and ideas with the healthcare team. These omissions may lead to additional felt losses compounding their grief and bereavement. It would appear that healthcare providers may lack understanding of parents’ loss and bereavement experiences.

Attached to the loss or impending loss of a child may be a plethora of emotions: grief, helplessness, guilt, and sadness. Parents, particularly mothers, may feel that when the focus is primarily on their child’s medical management, there is no space for them to express these feelings, and this can impact their developing sense of parenthood (Baum et al., 2012). The importance of building rapport, providing clinical updates, maintaining communication, and supporting parents through decisions following a shared decision-making approach is invaluable and can be considered best practice in the NICU (Caeymaex et al., 2013).

The broader NICU literature recognizes involving parents in each aspect of caring for a critically ill infant is essential and can be enhanced through family-integrated care (Waddington et al., 2021). This collaborative model integrates families as partners in the NICU healthcare team to effectively provide a structure that supports the implementation of family-centered care. Family-centered care in the NICU incorporates mutual respect and shared decision-making between the healthcare team and families, ensures quality psychosocial supports, promotes resource allocation focused on family wellbeing, and encourages families to directly deliver care to their infants, which can contribute to improved parent and infant outcomes related to bereavement (Sigurdson et al., 2020). However, goals of care discussions surrounding end-of-life and understanding how to best support bereavement may be challenging for parents and healthcare professionals as care needs to be individualized to each family’s situatedness (Lakhani et al., 2023; van Manen, 2021). Consequently, there is a need for research that gives voice to parental perspectives to inform the reflective practice of healthcare practitioners.

The purpose of this study was to elicit and represent NICU parents’ stories of goals of care decision-making and experiences that support their bereavement. Goals of care include decisions about specific treatments, the intensity of care, and planning for future care needs. Bereavement describes parents’ sense-making and living with the loss of their baby.

## Methods

This explorative study employed a narrative research design (Clandinin, 2006; Frank, 1995). Narrative inquiry understands stories as meaningful representations of people’s lives (Frank, 2006). In the telling and retelling of stories, people make sense of their past in the present. Stories are paradoxically both our own and shared as they both reflect our own sense-making and draw on the sense-making of others. To share a narrative means to articulate truths, find meaning in common experiences, and transform one’s identity in a social context (Weiss and Johnson-Koenke, 2023). The reflection taking place through narrative inquiry creates a story that can be filled with meaning, allowing the creation of self-identity while examining the underlying social and cultural contexts (Weiss and Johnson-Koenke, 2023).

### Narrative Ethics

At the core of narrative inquiry is an understanding of ethics whereby moral truths are founded and expressed in narratives (Frank, 2016; Murray, 1997). A narrative is not simply a story encompassing characters and events but rather a means of sense-making of human life and, specifically, moral life. We can understand moral sense-making by attending to how a narrator is situated within a story: a story’s beginning, plot, and ending; the relationships between characters of a story; and how a story is shared over time in the telling and retelling of a story (Frank, 2005, 2012). We seem to be able to accept those moral situations which we can talk about as “good” stories, not in the sense of entertaining stories but rather moral stories that we can recount without a sense of regret, despair, or wrongdoing. For this study, the stories of NICU parents were recognized to express normative aspects of NICU care that affect their living with the loss of their child. Following, aspects of care that speak to supporting bereavement are those which can be remembered, shared, or otherwise told; in comparison, those aspects of care that challenge bereavement are those which are recounted with sorrow, distress, or cannot be spoken of.

### Recruitment

Like others engaging in narrative research, we recruited participants to elicit their stories, following what could be described as a purposive approach (Guetterman, 2015; Joyce, 2015). The composition of our participants was informed by the desire to specifically seek out stories from individuals whose child had passed away in the NICU. We appreciate, however, that the stories told by the parents of children cared for in our institution may vary based on features such as parents’ age, race, gender, income, education, and spirituality, to name just a few distinguishing features of the storytellers. There are, of course, also varying features of hospitalized children as well as the healthcare team members involved. And, even in a single institution, there are variations in setting, time, materiality, and other existential features that are constitutive of lived experience (van Manen, 2019, 2021). In other words, it is impossible to elicit all of the possible storied perspectives of parents whose child had passed away in the NICU. So, we recognize in choosing a narrative approach, focusing on a relatively small volume of qualitative material, that we aimed for rich albeit tentative findings.

For this study, bereaved parents, who experienced a loss in the last 5 years, were approached by an intermediary through the Stollery Children's Hospital Aid for Symptoms and Serious Illness Support Team (ASSIST) Program. Exclusion criteria included a loss in the preceding 6 months or identified psychosocial distress. If consent to contact was obtained, a member of the research team contacted the potential participant by telephone or e-mail (based on the participant’s preference) to provide details of the study, review the study information, and obtain consent for participation as appropriate. None of the members of the research team had ongoing clinical involvement or responsibilities to the families.

### Data Collection

Interviews were scheduled virtually (based on the participant’s preference) to elicit the stories of parents who had lived the experience of losing a child in the NICU, recognizing that the parents’ present telling of their stories reflected their bereavement. In situations where both parents agreed to participate, we offered the opportunity to conduct interviews separately or together. While the interviews were oriented to participants’ stories, there were no specific initial or probing questions. Typically, the conversation started by asking participants: “Why don’t you tell me about the experience you and [your baby] had in the NICU.” The purpose of using an unstructured method was to ensure parents had the autonomy to share stories that were impactful to them regarding their experience with their child in the NICU. Participants were invited to share any or all aspects of the journey they had in the NICU as well as details on the pregnancy and post-NICU bereavement care. The interviews should be considered dialogic in the sense that the interviewer engaged in discussion with participants around their stories to elicit details and clarify understandings (Frank, 2005, 2012). The interviewer had no previous clinical contact with the families, specifically in their clinical care during their time in the NICU. Interviews were conducted from June to October 2022. Discussions were audio-recorded and transcribed verbatim with identifying features removed. Participants were also informed and provided consent for medical charts for their infants to be reviewed to contextualize demographic and clinical details after the interviews took place.

We recognize in many forms of qualitative research, “events, incidents, and experiences, not people per se” are the focus of data collection techniques (Sandelowski, 1995, p. 180). And we agree the number of participants needed for a qualitative study relates to multiple factors, including, but not limited to, the research method employed (Morse, 2000). For narrative approaches, the focus is on generating in-depth, textually rich storied data as a ground for analysis and interpretation (Joyce, 2015). In other words, perhaps it should be said that in narrative inquiry we are “sampling” stories not participants. Following, judgement regarding the adequacy of so-called “sampling” is not based on the number of participants but rather on whether “a new and richly textured understanding of experience” is achieved (Sandelowski, 1995, p. 183). We, therefore, implore the reader to judge the adequacy of our data collection based on their reading of our study findings, rather than the number of participants included.

### Analysis and Interpretation

Participants’ stories were read to explore normative aspects of their storied experiences that supported their bereavement. The stories that the parents recounted were read both holistically in the sense of the story of their child and family’s life and piecemeal as composed of fragmented stories, happenings, and anecdotes that stood out in their family’s journey. Qualitative data analysis software was not utilized. A typology of stories was used to thematize the interview material, reflecting the methodological commitments of dialogical narrative analysis (Frank, 2005, 2012). The analysis of the raw interview material, including the articulation of thematic aspects and selection of key quotations, was completed by authors Lakhani and van Manen. All listed authors contributed to the development of the narrative text. In the writing of the findings, we aimed to amplify and connect the themes from the participants’ stories. We include short, anonymized quotes with the use of pseudonyms to support the reader’s contextual understanding yet also preserve participants’ anonymity. The quotations can be read to provide supportive evidence for the validity of themes and to help convey contextual meanings of the findings of the study to the reader. Please note that data was drawn from all participant interviews, and we have included quotations from all participants in the Findings section of our manuscript. From a positionality perspective, the authors have clinical backgrounds in medicine, nursing, and ethics.

### Ethical Considerations

This study was reviewed by the University of Alberta Health Research Ethics Board. Before conducting interviews, participants provided consent for the study investigators to contact them regarding participation. They were made aware that the study was to uphold confidentiality and that no identifying information would be disclosed. All participants signed an informed consent document prior to the interview after receiving verbal and written information about the study. Participants were also informed that they could stop the interview at any time or refuse to answer any questions. When each interview ended, the interviewer attempted to understand the state of mind and emotional wellbeing of each parent. We aimed to achieve this by noting participants’ facial expressions, bodily posture, tone of voice, and other aspects expressive of their emotions. We also “checked in” with participants throughout the interview utilizing closed questions (e.g., are you ok to keep talking?) and open questions (e.g., how are you feeling after sharing your story?). Additionally, participants were advised they could contact the interviewer or study team if they had more information to share, needed psychosocial support, or wanted to meet again for any reason. Given the sensitive nature of the conversations and the experiences these parents have had losing a baby in the NICU, there could be a degree of distress felt by the participants in the telling of their stories. By providing the option to have multiple meetings rather than a single very long meeting, the interview was paced to the participants’ comfort. More so, this approach supported the participants to speak to their experiences in more comfortable pieces rather than an exhaustive whole.

### Participants

Seven parents, five mothers and two fathers, of five different infants who passed away in a Northern Alberta Canadian NICU program participated in the study. Specifically, three mothers were interviewed on their own, while two mother/father dyads were interviewed as a couple based on their requests. This number of participants supported a depthful engagement in their storied experiences. Like others, we would argue that saturation has differing relevance across traditions of qualitative inquiry (Saunder et al., 2018). Specifically, for narrative inquiry, saturation has limited relevance as experiences themselves are considered rich with a manifold of lived meanings that are variably disclosed through narrative descriptions. In other words, narrative studies cannot achieve saturation because there is always more meaning at the heart of experiences that in the telling and retelling of stories could yet be disclosed.

To provide additional context, participating parents had all experienced the loss of a child in the NICU within the preceding 5 years. They varied in their identified race and religion. Two families included parents that were newcomers to Canada. The duration of admission of the participants’ infants in the NICU ranged from 3 hours to 4 months. In three of the cases, there was an antenatal diagnosis of a significant life-limiting condition, whereas in the other two cases, diagnoses were made or confirmed after birth. Several syndromic diagnoses were made including, but not limited to, conditions of aneuploidy. Most of the infants underwent surgical interventions with the aims of prolonging their lives. A minority of families had a plan for comfort care in place before their children’s birth. All the infants passed away following a decision to withhold or withdraw life-sustaining medical interventions, such as mechanical ventilation. This demographic information is provided in narrative form to preserve the anonymity of the participants.

## Findings

Parents recounted stories and perspectives related to experiences with the NICU from before birth, to time in the NICU, to time after the death of their child. Narratives from parents contained many similar elements including having experienced an antenatal consultation, the use of medical interventions by the NICU team, and the loss of a child in the NICU setting. Variability, however, existed in the nature of each infant’s conditions, the number of subspecialists involved in decision-making, the need for transfer to different NICUs, the duration of admission, the chronological age of their child at the time of death, and, ultimately, the wishes of each family as they redirected care to comfort and palliation. From the parents’ experiences, we offer the following typology, recognizing that these normative aspects of care should not be read as thematically isolated but rather as converging on one another.

### Parents Recounted Stories of the Time They Had With Their Children...

“At that time, we only wanted to hold her . . . to have that time.”

Parents’ stories spoke to how their bereavement was supported by memories of being with their children. With relative ease, parents affectionately recounted watching over their children, holding their children, feeding their children, reading to their children, and other moments of being present for them in the NICU. Even in the context of illness, various critical care technologies, multiple healthcare providers, and so forth, parents’ stories of togetherness were celebrated as sources of meaning. Together is from Old English *togaedere*, “so as to be present in one place, in a group, in a body.”

“We sat the chair right beside the window, so we could hold him, and they brought in his bed. They had taken out his catheter, and then we went to the room, and I could just hold him. It was around 10 pm. And it was . . . it was a beautiful day. A beautiful day outside. There were no clouds in the sky. And the sun was setting, and we were just sitting there, the three of us.”

Stories included moments facilitated, affected, or otherwise mediated by unit technologies, healthcare provider practices, and other happenings in the NICU.

“They had a camera setup so even when we were at home, we could see him. So that was a really amazing thing. I think I had that camera open the entire time that we were at home.”

“And the nurse in the evening shift. She came in. And she said, “Can we give him a bath?” So, we gave him a sponge bath. And after that, I just said to her like, “Can I just hold him? Because I had never held him freely attached from tubes or anything.” So we just held him, and walked around the room. And we were in there for probably about a half hour just holding him, and then put him back down on the bed.”

Parents’ living with the loss of their children was storied by having had time together: time not just in the quantitative sense of a particular amount of time but rather time in the relational sense of having had the time to be with their child as their child’s parent, to come to know them, and for their child to know their family. In this way, parents’ stories of bereavement reflected their ability to “be there” to enact their felt need to care for their child in their child’s time of need, and their stories spoke to how the loss of their child left them with the memories they had of their time together as they experienced their role of being a parent, as an enduring and continuing bond.

“God let me hold my baby for fourteen days . . . I can still hold him, and he can hold my hand. I see him smile at me and his daddy, and he holds our hand, like this is my mama, you know what I mean? And when he, every day I [didn’t] leave my son from the moment he [was] born until his time he passed away . . . he knows his mama always be there for him, yeah, that’s it.”

In comparison, those moments of separation and disrupted togetherness were recounted with disease, discomfort, and distress. For example, parents reflected on having their baby experience an ambulance trip without having a parent present and those moments of leaving their baby in the hospital when they were discharged home from postpartum.

“So the first time he ever went outside, the first time he was ever in a vehicle, no parent was with him, and it’s really hard for me to remember because he was by himself, and I don’t like that.”

“It was hard definitely for when I was discharged from the hospital to leave him there and not come home with a baby in my arms.”

Regardless of whether the full diagnoses and extent of illnesses were known prior to delivery or not, parents’ stories expressed the depth and varied emotions they experienced when their child was taken to the NICU or away from them so soon after delivery. The etymology of bereavement is founded from the Old English *bereafian,* “to deprive of, take away by violence, seize, rob.” Following, stories of separation may be understood to have anticipated bereavement. In other words, at a time when parents were still fostering connections to their children, separation anticipated their longer-term living with loss as bereavement.

### Parents Shared Stories of the Healthcare Providers Doing What They Could and Should Do for Their Children . . .

Each parent wanted to know that the providers were doing what they could do and all that was going on with their child, to ensure that everything was being done and that nothing was being hidden. In this way, parents’ stories expressed the impact that healthcare providers had on their child’s NICU journey prior to their loss and the effects these relationships had on their bereavement. Generally, parents fondly recounted the conversations they had with members of the healthcare team and the relationships that were built over their time in the NICU. Although there were distinctions, nuances, and other individual aspects of valued communication, all parents shared stories of appreciated timely and honest communication supporting confidence and trust in the healthcare team.

“The communication piece [with hospital staff], I would say through it all, was definitely above and beyond than what we would ever expect.”

“And whatever questions I had they gave me the answers. And they made me feel that they were taking care of him the best that they could.”

Tailoring care to each family’s beliefs of what was in their child’s interests based on their individual, family, and cultural background was something that parents described as invaluable. However, their stories also reflected how consideration needed to be given to varied understandings of their beliefs that may or may not align with healthcare providers’ interpretations of them.

“I [had] a proper Muslim doctor tell me to take off oxygen from my son, because it doesn’t help him . . . [that] he will not survive. But in my beliefs, doctors come to save people, they don’t try to give up [on] the patient’s life . . . but this doctor, [a] proper Muslim doctor tells me, even [he] tells me, he wants to bring my Imam.”

Providers doing what they should do is related to notions of confidence and trust. While the etymologies of confidence and trust intersect, distinctions can be made. Confidence is based on evidential skills, knowledge, and qualifications. In comparison, trust has a normative quality, which may or may not be based in perceptions of skillfulness, reflecting how we feel toward the moral character of another. Following, not only did parents positively recount doctors, nurses, and other healthcare providers’ expertise but also that they appropriately acted in the interests of their child. More so, parents felt like they were part of the medical team, and that their opinions were acknowledged and valued. Parents’ stories recognized that they knew their child in a different, and arguably better, way than the healthcare team—allowing parents to feel they could and should contribute to the care of their children. Moments, whereby parents were less engaged in both extraordinary and everyday moments of care, were recounted as missed opportunities, at times with regret and sorrow.

“I think I had only held him on one occasion before he passed away . . . Or they let us and gave us the opportunity to help like change his diaper, even though he had a colostomy bag and catheter anyways . . . We did have those options, but I wish it would have been more, offered to us again and again.”

Although the medical interventions undertaken in support of a child’s condition were not always successful, parents’ stories highlighted how having done all that could and should be done supported parents’ living with their end-of-life decisions. In addition, the storied accounts of the medical team’s exhaustive efforts, not to change the outcome or alter the prognosis for their child but to ensure each infant was comfortable and supported, were valued.

### Parents Told Stories of Living With Uncertainty, Ambiguity, and Indecision...

Uncertainty is an inherent challenge of prognostication in the NICU, and parents’ stories reflected this difficulty when receiving information about their children’s diagnoses and prognoses and facing subsequent decision-making. Nothing in medicine is ever one hundred percent certain, and this not only troubles the healthcare team as they attempt to support parents’ decision-making on how to proceed, but it can also cause a significant mental burden for families as they attempt to make decisions that will impact the fate of their child’s life.

“It would have been [nice] to know the prognosis like way in advance and know what we’re looking at. Because it was like, it was frustrating and it was hard like emotionally and physically, everything, just not knowing what’s going on. Right? And people want to reach out, they want to ask, we just didn't know anything, right?”

Parents recounted with sensitivity and, at times, difficulty, stories of receiving unexpected news that contributed to further ambiguity of their child’s condition. The etymology of ambiguity is from Old French *ambiguite*, encompassing aspects of doubt, indecision, and hesitation. An ambiguous situation is open to varied interpretations in its equivocality. While uncertainty was resolved in time for some of the parents, for others, bereavement encompassed living with unresolved ambiguity. This was difficult and burdensome for families.

“I remember like when they brought him back and then we’re like, oh his lungs looked irregular like, he knew like something else was not good . . . there’s something else that’s going on . . . to us he looked like perfect . . . And then the condition that he was diagnosed with is super rare.”

Stories of uncommon, undifferentiated, or unknown diagnoses wore on families as they lacked an ending. And yet, even in the face of uncertainty and ambiguity, some parents reflected on specific events or even just a moment when they realized that it was time to change the course of their infant’s care and accept palliation as an appropriate next step.

“They actually said to us that he is not strong enough to have that surgery. And when we heard that we said, we just thought, okay, well, the decision that we can make for him is to let him be in peace as compared to continuing to suffer in the hospital like he is.”

Parents reflected on the difficulty in making this decision; most did not seem to regret their choice, but some continued to question, in recounting their stories, what would have happened if they had one or two different choices along their journey.

“I think there’s always going to be that what if question of, what if we decided to not put them in comfort care? Obviously, he would still be here living with [his] syndrome. But like, how sick would he be? Would he be okay? Would he need specific medication? What would have his potential life expectancy be?”

### Parents Shared Stories That Expressed Values of Caring . . .

Parents’ stories spoke of the difficult and emotional moments they experienced in the NICU. These onerous moments were at times alleviated by, or at least eased by, the caring actions of the healthcare team. Specific bedside nurses stood out as key characters in the parents’ stories who were supportive in both day-to-day care and those exceptional events in their children’s lives. Virtues of honesty, compassion, trust, resourcefulness, and patience were recognized in the skillful manner that these healthcare providers worked with them.

“Nadine was definitely a rock that day. Whatever we needed she was there.”

“Stephanie literally thought of everything that we didn’t. She had all his stuff packed, like she helped organize, she helped just get everybody just be there and fill in everything that we needed. They moved a couch for us at one point.”

Parents’ stories emphasized how the close relationships that were built over time in the NICU created an outlet of support within the medical team. Being able to release the mental and emotional burden to someone you trust is something that was storied as highly valuable to parents. Familiar personnel that understood not only their child’s medical history but also their family’s circumstances, their wishes for their child, and their coping abilities were fondly described by several parents through their stories. For example, one family spoke highly of the primary care team that they chose for the infant and the role this had in supporting them through the difficult moments in their child’s course.

“[The neonatologist] understood it was important to us to have him actually physically talk, instead of getting it third party . . . so he made times on weekends to meet us up at the [hospital] or calls at night . . . and that made everything better.”

Of all the encounters parents reflected on through their stories, receiving direct, open, and honest updates expressed through care about their children’s clinical status and prognosis often helped parents cope with the news they were receiving and eased the mental burden of decision-making. Parents’ stories spoke to the value of candid and timely conversations that necessitated a caring relationship of trust.

‘Hearing that as a parent, it’s very, very hard. But we also put our complete trust into the doctors and the nurses and the people who are caring for him to do the best that they can do.”

### Parents Talked About What Their Child’s Life Was Like . . .

Regardless of the number of days, hours, or minutes spent with their child, parents described the significance of their child’s life and the experiences their child was afforded before their death. While the time they had together was highly valued, parents’ stories expressed the tension between affording time together and potentially prolonging pain, suffering, or discomfort.

“I would go to touch his head, and he would kind of like, I don’t know what the word is, but make a face that that wasn't comfortable for him. It was painful.”

“. . . as we got like further along and I remember like towards the end he was so swollen and just looked like sick and uncomfortable.”

“. . . is that like humane? . . . [to] sedate him so much? So he doesn’t pull out his tube, and then tie him—like have him restrained.”

The events leading up to the redirection of care toward palliation, withdrawal of life-sustaining medical interventions, and the resulting final moments of their children’s lives were often recounted in great detail, illustrating the impact these events had on parents over time. Perceptions of suffering often lead to a change of heart and shifting treatment goals toward comfort care.

“And then, so Jeff and I came home and talked. We spent all day Thursday up there going through everything. And then we came home again and decided that . . . We can’t see him suffer anymore.”

“They had asked us if we wanted to wait . . . but Aliyah was just, at that point, suffering, and you could see it.”

Parents’ stories illustrated the difficulty in deciding to redirect the care of their infant as one of the toughest decisions they have had to make in their lives; however, seeing their child at peace after their final moments validated these decisions and provided them with comfort.

“Yeah. And I think the one thing that helped us—well I guess not helped us, but kind of just reassured [us] was time, was that he didn’t last long without the breathing tube.”

“I think like, she wasn’t kind of like gasping for air . . . so she looked at peace.”

### Parents Shared Stories of Living With Loss . . .

Often healthcare providers do not have a chance or fail to think about the moments just after a baby passes away in the NICU; however, through parents’ stories, it is clear that these times are some of the most challenging for parents as they come to the realization that they will be leaving the hospital without their baby. The time spent in the NICU working toward comforting and supporting their child is no longer part of their daily life—their world as they know it will be entirely different from that point forward.

“After she had passed away, I didn’t know what was next. I have no idea like what happens now. And she was given to me. She stayed with us in the room. Like she was kind of like put in kind of like a cold crib. And then like, I was like, my husband was saying like “I’m arranging for burial.” And I was like what do we do? Do we just go out? Do we just—I don’t know.”

It is more than knowing the next steps of identifying a funeral home, planning a service, and so forth. Families’ stories expressed the lasting impressions they were left with of their children and the care they received.

“There’s one thing that I regret. And that's when I was leaving, I just took one more glance into the room. And it’s a very weird being and seeing situation, where for the past 3 weeks, your son is in a room and he’s got these tubes, he’s got these monitors, he’s got everything and everyone that’s looking after him. There’s always somebody in the room watching him. And then when you leave, he’s the only one in the room. There are no monitors on, he’s sleeping in his bed, and that’s all you see. And that’s one memory that I wish I didn’t have to experience. It’s really painful to think back to.”

Parents’ stories shared the various emotions that arise when living with loss over the coming days, weeks, months, and years including sadness, emptiness, guilt, grief, regret, uncertainty, and, in some cases, acceptance. Parents described these emotions as connected, interspersed, and ongoing.

“And like, but I think I knew that deep down, but it’s still, I still like will go back and forth and be like, you know what, I wish I could have . . . And that’s just how I am in my daily life.”

Finding appropriate support, from both personal contacts and health professionals, was described through parents’ stories as being appreciated and helpful in not necessarily overcoming their loss but learning to live with it. Through follow-up with the hospital team, having a chance to ask questions and seeking support from a bereavement counsellor long-term, parents’ stories spoke of the ability to process grief and learn to live with it individually as well as within the context of their relationships with one another.

“[The bereavement counsellor] helps a lot with like the milestones. So, like when we reached [his] first birthday, the anniversary of his passing, and like going through the holidays and that . . .”

“What was really better for us was bereavement counseling that was offered to us through the [hospital]. Because she helped us understand the way that each other was grieving.”

Regardless of the duration of their children’s lives, parents’ stories spoke to how their bereavement, both in the short and long term, has been supported by tangible memories they could keep, display, or share with loved ones. With positivity and happiness, parents’ stories expressed their love for their children while describing or presenting these mementos as a chance to share their quality time with their infants through long-lasting physical objects. These objects and the memories they sparked were recounted as continuously cherished by families.

“They brought us a little memory box, with his prints in it, the card with his name on it, his wristbands, he had a comb, his nose apparatus, breathing things, just like the personal things that they could put into the box for him . . . it was hard, definitely. But they personalized it . . . and that was really special.”

## Discussion

This study sought to explore NICU parents’ stories of goals of care decision-making and experiences that helped support their bereavement. Through interviewing a diverse group of parents who have gone through such an experience in a Northern Alberta Canadian NICU, we were able to identify areas of “value” (moral sense-making). We learned how their stories reflected on individual aspects of the importance of having meaningful time with their children, maintaining direct and frequent communication with the healthcare team, acknowledging uncertainty, and emphasizing the positive impact of compassion as helpful to navigate their bereavement. The storied experiences described by parents provide unique insights for healthcare practitioners in supporting bereavement in the NICU as individualizing support is an essential component to family-centered care.

Broadly, the stories told by bereaved parents and the unique experiences they had in the NICU parallel the values and recommendations for bereavement care seen in the neonatal literature, thus unveiling similar strengths and weaknesses in neonatal bereavement care (Lakhani et al., 2023). For example, parents’ stories emphasized the importance of participating in their child’s care, whether it be changing diapers or assisting with feeds, as well as the opportunities they had to hold their infant and create an emotional connection. This corresponds with several studies where early involvement in care and taking on the parenting role is an essential method of support for parents of critically ill children (Abraham and Hendriks, 2017; Armentrout, 2009; Baughcum et al., 2017; Currie et al., 2016; Thornton et al., 2020). Recognizing that early physical bonding experiences can have formative impacts on parents as they navigate the uncertainty of their infant’s prognosis, healthcare practitioners should attempt to support early experiences such as skin-to-skin contact, handholds, and other physical contact whenever safe and appropriate to do so to limit early physical separation and the associated burdens. Similarly, the appreciation parents felt for photographs and tangible mementos to maintain their child’s legacy is one that has been well-described (Alexander, 2001; Cortezzo et al., 2015; Kymre and Bondas, 2013; Levick et al., 2017; Oreg, 2020). Parents’ stories alluded to how the care they received in the NICU contributed to sense-making in the time following the death of their infants, and some explicitly described the importance of tangible items in assisting that healing process. Being able to make sense of the experienced loss can help parents navigate grief and move toward acceptance (Keesee et al., 2008). We hope the reader also appreciates that our research uniquely offers a reading of possible storied experiences of the loss of a child in the NICU. Healthcare providers may benefit from a reflective awareness of what this experience may be like to inform their clinical practice, recognizing that each child and family are unique. This kind of understanding resists summarization as it is actualized in specific healthcare encounters with families.

Bereavement is a long-term process that benefits from tactful and compassionate communication, shared decision-making, and individualizing care to help parents grieve, find closure, and move toward acceptance after loss (Clarke and Booth, 2011; Cortezzo et al., 2015; Currie et al., 2019; Oreg, 2020). It is important to note that the stories told by parents did elicit some unexpected discrepancies in methods of support that have been previously identified in the literature. For example, while the benefits of cultural relatability and alignment have long been well-described (Abdel Razeq & Al-Gamal, 2018; Armentrout, 2009; Rosenbaum et al., 2011), it is important to consider individual understandings and interpretations of cultured values and beliefs, which may not have such a positive impact. Although a religious or cultural similarity may exist between a family and members of the healthcare team, priorities and values regarding patients’ care may not be aligned, and this can cause additional stress and turmoil for parents. These discrepancies help emphasize the importance of individualizing bereavement care to each family and their sociocultural circumstances.

Though individualizing care is an essential component to providing effective bereavement care, parents’ stories wholly illustrated the impact healthcare providers had on their bereavement in the short and long term. There are several supportive measures, as seen in the literature, that healthcare practitioners *should* generally provide that help support families—frequent communication with the healthcare team, encouraging parents to participate in daily rounds, involving ancillary services, and supporting parents to spend quality time with their infants (Clarke and Booth, 2011; Cortezzo et al., 2015; Currie et al., 2019; Oreg, 2020; Sigurdson et al., 2020). This study illustrated additional ways healthcare practitioners *could* enhance family-centered care and provide parents with other realms of support in their bereavement. Some techniques gathered through parents’ stories contribute new understandings to the existing literature on neonatal bereavement support, and these include offering additional points of contact to discuss medical updates and decisions, appointing a primary care team to take the lead in their child’s care, and providing guidance on steps to take in the immediate moments after death. These added supportive measures, not as prominently seen in the existing literature but seen as highly valued as recounted through parents’ stories, noticeably illustrate the variety of ways healthcare practitioners can contribute to not only the care of a critically ill child but also assist in the support of their families as they navigate their grief and bereavement during and after their loss. These themes found through parents’ stories can ultimately be used to further develop healthcare providers’ reflective practice and training techniques for healthcare professionals to help optimize bereavement support and communication in the NICU.

Limitations of this study relate to the number and diversity of parents interviewed from a single institution. All the participants had a partner to share these experiences with. Some stories recounted the comfort and support that their significant other provided both during the critical decision-making period and during their bereavement. Future work should consider the experiences of single parents, parents affected by poverty, siblings and extended family, Indigenous families, and other groups that have traditionally not been engaged in empirical qualitative NICU research studies. The small number of participants from a single geographical area may limit the generalizability of the gathered stories; however, the ability to obtain in-depth reflections from the participants elicited important insights and unique contributions to the current literature.

From exploring bereaved parents’ stories, we are offered understanding into aspects of NICU care that may support their bereavement. Supporting parents to have meaningful time together with their children, orienting care toward what we can do for a child, acknowledging and dealing with uncertainty as able, focusing on values of caring, and fore-fronting what a child’s life is like may ultimately support parents living with the loss of their infant. At the same time, we may recognize bereavement as “an ever-open wound” (Sartre, 1998, p. 624). Care for these families does not end with discharge from the NICU. Instead, they have ongoing needs that may benefit from coping strategies and work toward acceptance as opposed to dissolving the emotions surrounding grief and loss.
